# Influence of soil properties on the bioaccumulation and effects of arsenic in the earthworm *Eisenia andrei*

**DOI:** 10.1007/s11356-015-4659-4

**Published:** 2015-05-24

**Authors:** A. Romero-Freire, F. J. Martín Peinado, M. Díez Ortiz, C. A. M. van Gestel

**Affiliations:** Department of Soil Science, University of Granada, Avd. Fuente Nueva, 18002 Granada, Spain; NanoHealth & Safety group, R&D department of LEITAT Technological Centre, Carrer de la Innovació, 2, 08225 Terrasa, Barcelona Spain; Department of Ecological Science, Faculty of Earth and Life Sciences, VU University, De Boelelaan 1085, 1081 HV Amsterdam, The Netherlands

**Keywords:** Arsenic, Bioavailability, Soil properties, Earthworm accumulation, Toxicity

## Abstract

This study aimed at assessing the influence of soil properties on the uptake and toxicity effects of arsenic in the earthworm *Eisenia andrei* exposed for 4 weeks to seven natural soils spiked with different arsenic concentrations. Water-soluble soil concentrations (AsW) and internal As concentrations in the earthworms (AsE) were greatly different between soils. These two variables were highly correlated and were key factors in earthworm toxicity response. AsW was explained by some soil properties, such as the pH, calcium carbonate content, ionic strength, texture or oxide forms. Toxicity showed a clear variation between soils, in some cases without achieving 50 % adverse effect at the highest As concentration added (600 mg kg^−1^). Nevertheless, soil properties did not show, in general, a high relation with studied toxicity endpoints, although the high correlation with AsW could greatly reduce indirectly As bioavailability and toxicity risk for earthworms. Obtained results suggest that soil properties should be part of the criteria to establishing thresholds for contaminated soils because they will be key in controlling As availability and thus result in different degrees of toxicity.

## Introduction

Arsenic is a metalloid which is found at widely varying concentrations in different environments (Kabata-Pendias and Pendias 2001). Currently, its presence is associated with natural and anthropogenic sources, and in many areas, its environmental levels in water, sediments or soils are of major concern due to its potential adverse health effects (Nriagu et al. 2007).

To determine the reference levels of metals in soils, it is necessary to know their contents under natural conditions (soil background) (Martín et al. 2012). Arsenic background concentration in soil is highly variable as it depends on the initial concentration in the parent material, natural geochemical cycles and soil type (Díez et al. 2007). The bioavailability and therefore the toxicity of metals to organisms in terrestrial ecosystems are largely controlled by soil properties (Sheppard and Evenden 1988). The solubility of arsenic and therefore also its toxicity are known to be strongly controlled by soil properties and constituents (Martín Peinado et al. 2012). The most important variables affecting As availability in soils seem to be the organic carbon content, pH, ionic strength of the soil solution, iron oxides and cation exchange capacity (Romero-Freire et al. 2014; Song et al. 2006).

Evaluation of the effects of contaminants in soils has become a priority for OECD member countries (Saint-Denis et al. 2001; Arnaud et al. 2000). Most environmental protection policies are based on guidance based on total concentrations of a particular element or compound in soil, referring to land-use type but not to the soil type. This creates a large variety of soil quality criteria for metals in different countries. For example, the intervention levels for As in agricultural soils in Europe range from 10 mg As kg^−1^ soil in the UK (Barth and L’hermite 1987) to 50 mg As kg^−1^ soil in the Netherlands (NMHPPE 1994).

The accumulation of trace elements from soil to biota has been studied extensively for many species (Díez-Ortiz et al. 2010; Nahmani et al. 2007). Earthworms are more susceptible to metal pollution than many other soil invertebrates (Spurgeon and Hopkin 1996; Bengtsson et al. 1992). Furthermore, earthworms have a number of characteristics (large size, behaviour and high biomass) which make them highly suitable animals for use as bioindicator organisms for determining the toxicity of chemicals in soils (Arnaud et al. 2000; Callahan 1988; Goats and Edwards 1988; Bouché 1992). Consequently, they have been adopted as standard organisms for ecotoxicological testing by the European Union (EEC 1984), with *Eisenia andrei* commonly used as the test species not only in standardized toxicity tests (OECD 1984) but also in bioassays to assess the toxicity of field-contaminated soils (Fleuren et al. 2003; Cortet et al. 1999).

To determine toxicity guidance values, usually artificially contaminated soils are used, which may differ in composition from in situ contaminated soil. As a consequence, laboratory experiments tend to overestimate the solubility and availability of metals compared to field-contaminated soils (Smolders et al. 2009; van Gestel et al. 2012). The toxicity level defined by laboratory studies commonly overestimates the effects, which can lead to high strict safety thresholds for the environmental risk assessment (Romero-Freire et al. 2014).

The aim of this study was to determine the effect of soil properties on the bioavailability of arsenic by measuring its uptake and toxicity using the earthworm *Eisenia andrei*. The earthworms were exposed to a wide range of soils with contrasting properties spiked with different arsenic concentrations. This study is part of a broader project that involves assessing the toxicity of arsenic to different organisms for the purpose of establishing guideline values to improve the existing regulation regarding soil pollution in Spain (Romero-Freire et al. 2014).

## Materials and methods

### Soils

Seven soils with different properties, representing most of the main soil groups in Spain, were selected (Table 1). The main parameters analysed were pH (soil to water or soil to 0.1 M KCl in a ratio 1:2.5), ionic strength of the soil (I) derived from the electric conductivity according to Simón and García (1999), calcium carbonate content (CaCO_3_), organic carbon content (OC), available phosphorous content (P), water holding capacity (WHC), available water (AW) calculated from differences in moisture contents at the field capacity (h33) and the wilting point (h1500), texture, cation exchange capacity (CEC). These properties were determined according to official methods of analysis (M.A.P.A. 1994). Moreover, concentrations of free and amorphous iron, aluminium and manganese oxides were analysed according to Holmgren (1967) and Schwertmann and Taylor (1977), respectively.

**Table 1:** Main properties of the soils used to assess the influence of soil properties on the uptake and effects of As in earthworms

Soil	As^a^ (mg kg^−1^)	pHH_2_O (1:2.5)	pHKCl (1:2.5)	I (mmol L^−1^)	P (mg kg^−1^)	CaCO_3_ (%)	OC (%)	Clay (%)	Silt (%)	CEC (cmol^+^ kg^−1^)	Al_d_ (‰)	Al_o_ (‰)	Fe_d_ (‰)	Fe_o_ (‰)	Mn_d_ (‰)	Mn_o_ (‰)
H1	15.5	7.96	7.63	7.3	8.3	37.1	5.43	23.6	42.3	21.4	2.40	1.17	19.0	0.68	0.54	0.06
H2	9.07	8.67	8.11	2.0	bdl	72.4	0.42	11.8	46.8	9.83	1.10	0.31	8.67	0.20	0.13	0.02
H3	3.39	8.79	8.24	1.7	bdl	92.3	0.38	7.70	64.0	2.94	0.60	0.15	3.29	0.01	0.01	0.00
H4	16.2	6.74	5.80	0.9	6.5	bdl	0.61	19.0	24.3	9.91	1.90	0.38	17.9	0.52	0.32	0.18
H5	12.3	7.20	6.72	13	28.1	bdl	8.22	23.8	33.3	25.9	1.90	0.50	19.4	0.65	0.85	0.41
H6	4.39	5.87	4.58	0.5	1.1	bdl	0.49	8.31	21.2	3.83	0.90	0.27	7.77	1.00	0.15	0.09
H7	25.7	7.03	5.86	1.6	bdl	0.92	0.66	54.7	15.3	15.5	5.10	0.73	82.6	0.78	0.13	0.03

Free forms of Al_d_, Fe_d_ and Mn_d_ and amorphous forms of Al_o_, Fe_o_ and Mn_o_

*I* ionic strength, *P* available phosphorus content, *OC* organic carbon content, *CEC* cation exchange capacity, *bdl* below detection limit

^a^Total As background

Soils were contaminated in the laboratory with increasing concentrations of sodium arsenate (Na_2_HAsO_4_ · 7H_2_O) according to the reference values proposed by the Junta de Andalusia (Aguilar et al. 1999) for agricultural, natural and industrial use (50–100–300 mg As kg^−1^, respectively) and adding one more level to create a worst case scenario (600 mg As kg^−1^). Furthermore, an uncontaminated level (control) was included. Contamination was performed by spiking samples of 500 g of soil (dry weight) with aqueous As solutions (*n* = 3). After spiking, the soils were moistened to 60 % of their WHC and incubated for 4 weeks at 25 ± 1 °C and 60 % air humidity, with a light to dark cycle of 10:14 h. Soil moisture content was checked and, if needed, readjusted weekly. The incubation period chosen allows stabilization of the arsenic added and was based on similar studies by other authors (Romero-Freire et al. 2014; Tang et al. 2006; Fendorf et al. 2004). After the incubation period, a saturated extract was prepared with a soil to water ratio of 1:1 and was stirred for 24 h; then, a soil solution was obtained by extraction with a 10-cm Rhizon MOM and analysed for pH, electrical conductivity, and water-soluble As concentrations (Romero-Freire et al. 2014).

### Earthworm toxicity testing

Earthworms of the species *E. andrei* were supplied by Lombricor SCA (Córdoba, Spain). Earthworms were cultured at 20 °C in a substrate of soil with high organic matter content, peat and abundant horse manure free of any pharmaceutical. Before the start of the exposures, adult worms with well-developed clitella were selected with an average weight of 0.50 ± 0.08 g.

The earthworm toxicity tests followed OECD guideline 222 (OECD 2004), including a 4-week exposure period of adult animals. Three replicate test containers were used for each arsenic concentration and control, containing approximately 500 g soil (dry weight equivalent). Ten adult earthworms were added to each test container after being gently cleaned on moistened paper towels and weighed. To feed the worms, 25 g of horse manure:distilled water (1:4 ratio) was added to each container. The containers were kept in an incubator chamber at 20 °C with 12 h of light per day. Container weights were monitored weekly to maintain moisture content, and additional food was added when required.

After 4 weeks, test containers were emptied into a tray, and surviving adults were collected by hand sorting, cleaned and weighed. Surviving earthworms were placed on moist filter paper for approximately 24 h to void their gut contents, following Arnold and Hodson (2007). After weighing, they were freeze-dried and stored for analysis. Soils, which contained cocoons, were returned to their respective containers and incubated for another 4 weeks controlling the water content weekly. After this period, the containers were placed in a water bath at 60 °C, forcing juveniles to emerge to the surface, where they were counted.

### Arsenic analysis

Total arsenic background concentrations in soils and earthworm tissues (AsE) were determined after digestion in a mixture of concentrated HNO_3_:HCl (4:1). Water-soluble As concentration in soil (AsW) was determined from soil:water extracts (1:1 ratio) after 24 h equilibrium with shaking (Romero-Freire et al. 2014, 2015; Fotovat and Naidu, 1998). In all cases, As was measured by inductively coupled plasma-mass spectrometry (ICP-MS) in a ICP-MS NexION 300D spectrometer. Instrumental drift was monitored by regularly running standard element solutions between samples. For calibration, two sets of standards containing the analyte of interest at five concentrations were prepared using rhodium as an internal standard. Procedural blanks were included for estimating the detection limit (3 × *σ*; *n* = 6) which was <0.21 μg/L for As. The analytical precision was better than ±5 % in all cases. The accuracy of the method was confirmed by analysing standard reference material SRM2711 Montana Soil (US NIST 2003). Total As concentrations (AsT) in spiked soils were checked using portable X-ray fluorescence (PXRF) (Martín Peinado et al. 2010), and measured concentrations ranged between 80 and 97 % (average ± SD 92 ± 5 %; *n* = 34) of the nominal values.

### Data analysis

Soil-water partition coefficient (Kp) was calculated as the ratio of the total As concentration in soil (AsT in mg kg^−1^ dry soil) and the water-soluble As concentration (AsW in mg L^−1^) and expressed as litres per kilogramme (Blaser et al. 2000). Biota-soil accumulation factors (BSAF) for the uptake of As in the earthworms after reaching the steady state (OECD 317, 2010) were calculated by dividing concentrations in the surviving animals (AsE; in mg As kg^−1^ earthworm dry weight) by total concentrations in the tested soils (AsT; in mg kg^−1^ dry soil) (Peijnenburg et al. 1999). Biota-water accumulation factors (BWAF) were calculated by dividing AsE by water-soluble As concentrations (AsW; in mg L^−1^) (Peijnenburg et al. 1999). Lethal concentrations causing 10 % mortality (LC_10_) and effective concentrations causing 50 and 10 % reduction of juvenile production (EC_50_, EC_10_) and their corresponding 95 % confidence intervals were calculated by fitting a log-logistic dose-response model to the data (Doelman and Haanstra 1989) for soil samples which showed a dose-response relationship with AsT, AsW and/or AsE.

To assess As toxicity for earthworm exposition, mortality (M) was calculated as the percentage of worms that died during the 4-week exposure period for each soil and treatment in relation to the control; weight variation (W) was calculated as the percentage of variation in the surviving earthworms recovered after 4 weeks in relation to the initial weight and recalculated in relation to the control soils; juvenile production (J) was calculated from the number of juveniles produced per worm per week and expressed as the percentage in relation to the control.

Normal distribution of the data was verified with a Kolmogorov-Smirnov test. Significant differences were determined by ANOVA, and multiple comparison analyses were performed with Tukey HSD test (*p* < 0.05). To study the influence of soil properties on accumulation and toxicity of arsenic by *E. andrei*, Spearman’s correlation analysis and principal component analysis (PCA) after varimax rotation were applied to discriminated different groups of variables according to statistical similarities of the normalized dataset. All these analyses were performed with a confidence level of 95 % by using SPSS v.20.0 (SPSS Inc. Chicago, USA).

## Results

### Water-soluble arsenic concentrations and soil properties

Water-soluble As concentrations showed a dose-related increase and were significantly higher (*p* < 0.05) at the highest As concentrations added (>300 mg kg^−1^) for all studied soils and also differed between the tested soils (Table 2). The lowest water-soluble As concentration was found in the iron-rich soil H7 (red Mediterranean soil) with 3.34 mg As kg^−1^ soil extracted at the highest concentration tested (600 mg As kg^−1^ soil). Soils rich in organic carbon (H1 and H5) also showed low water-soluble arsenic concentrations, with extractions of 82.9 and 106 mg As kg^−1^ soil, respectively, at the highest exposure concentrations. In soils H4 and H6, which are slightly acidic, non-carbonated and had low ionic strength, 116 and 118 mg kg^−1^ of the As were water-soluble at 600 mg As kg^−1^, respectively. Soils with the highest CaCO_3_ content and basic pH (H2 and H3) had the highest water-soluble arsenic concentrations (271 and 337 mg As kg^−1^ soil, respectively). Table 2 also includes the partition coefficient (Kp) for As in the different soils. Kp showed a significance difference among treatments in the different studied soils. Higher Kp values appeared in control soils; however, this was not the case in soils H4 and H7, where high values appeared at the lower As treatment levels.

**Table 2:** Mean water-soluble arsenic concentrations (AsW; mg As kg^−1^ soil), partition coefficient calculated as the ratio of the total As concentration in soil and the water-soluble As concentration expressed as milligrammes per litre As (Kp; L kg^−1^), arsenic concentrations in earthworms (*Eisenia andrei*) after 4 weeks exposure (AsE; μg As g^−1^ dry body weight), biota-soil accumulation factors (BSAF; kg soil kg^−1^ earthworm); biota-water accumulation factors (BWAF; L kg^−1^ earthworm)

Soil	As nominal	AsW	Kp	AsE	BSAF	BWAF
	mg kg^−1^	mg kg^−1^	L kg^−1^	μg g^−1^ d.w.	kg soil kg^−1^ worm	L kg^−1^ worm
H1	0	0.023a	689b	32.5a	2.06a	1376b
	50	0.49a	134a	113ab	1.73a	228a
	100	1.78a	64.9a	170ab	1.50a	94.9a
	300	22.4b	14.1a	504b	1.60a	22.5a
	600	82.9c	7.5a	972c	1.56a	11.6a
H2	0	0.007a	1295d	67.9a	7.49b	9710b
	50	0.96a	61.8c	96.7a	1.63a	103a
	100	6.45a	16.9b	182a	1.66a	28.2a
	300	85.3b	3.63a	326ab	1.07a	3.83a
	600	271c	2.27a	525b	0.87a	1.96a
H3	0	0.010a	339d	26.01a	7.67c	2600b
	50	6.56a	8.17c	482b	9.03bc	73.9a
	100	29.5a	3.5b	594bc	5.73b	20.2a
	300	153b	2.00a	762c	2.53a	5.0a
	600	337c	1.80a	ns	ns	ns
H4	0	0.077a	218b	18.8a	1.17a	258a
	50	0.12a	579c	278ab	4.20b	2482b
	100	0.57a	205b	516c	4.43b	923a
	300	23.1b	13.8a	667c	2.11a	29.4a
	600	116c	5.33a	718c	1.16a	6.2a
H5	0	0.014a	1022b	28.35a	2.33b	2311b
	50	1.05a	59.5a	600b	9.63d	573a
	100	3.44a	32.6a	688bc	6.13c	200a
	300	29.3b	10.6a	768c	2.43b	26.2a
	600	106c	5.83a	609b	0.99a	5.83a
H6	0	0.010a	439a	32.2a	7.33a	3219c
	50	0.36a	154.2b	606b	11.1b	1743b
	100	1.08a	97.9c	740b	7.09a	698a
	300	23.1b	14.7d	ns	ns	ns
	600	118c	5.13d	ns	ns	ns
H7	0	0.010a	2566a	23.3a	0.93ab	2334ab
	50	0.013a	6305b	64.2a	0.83a	5426c
	100	0.023a	5585b	122a	0.97ab	5411bc
	300	0.28b	1189a	540b	1.67ab	1953b
	600	3.34c	188a	1019c	1.93b	362a

As nominal: total arsenic added to the soil in milligrammes per kilogramme. See Table 1 for soil properties. Lowercase letters show significant differences among treatments for each soil (Tukey HSD test, *p* < 0.05)

*ns* no survival

**Fig. 1:**
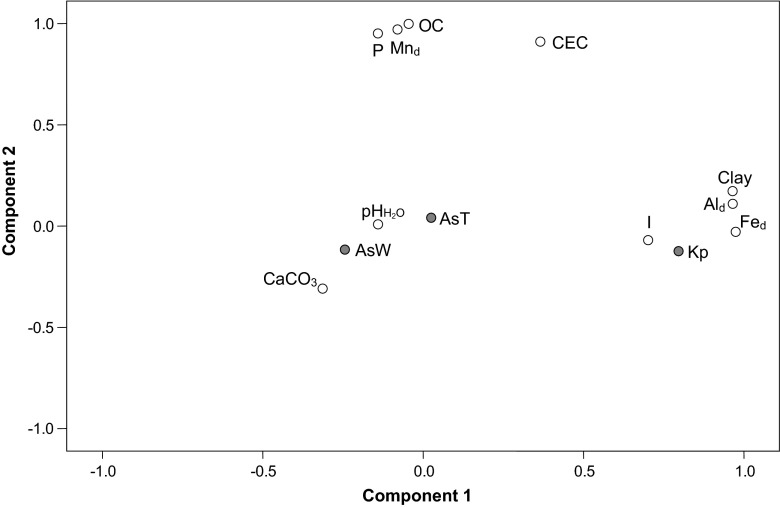
Principal component analysis (PCA) after varimax rotation including As forms (*AsT* total arsenic concentrations, *AsW* water-soluble arsenic concentrations), partition coefficient (*Kp*) and the main soil properties (*I*: ionic strength; *Fe*_*d*_, *Mn*_*d*_ and *Al*_*d*_: free forms of iron, manganese and aluminium; *CEC*: cation exchange capacity; *OC*: organic carbon content; *P*: available phosphorous). Accumulate variance explained for component 1 = 32.8 % and for component 2 = 62.3 %

The factorial analysis expressed as a principal component analysis (PCA) between AsT, AsW, Kp and some of the main soil properties showed that 62.3 % of the variance was explained by two components (Fig. 1). The arsenic forms (water-soluble and total) were grouped with pHH_2_O and CaCO_3_ content. In component 1, Kp was strongly related with clay content, ionic strength (I) and free forms of Al and Fe, while in component 2, phosphorous content (P), free forms of Mn, organic carbon (OC) content and cation exchange capacity (CEC) were grouped together.

### Arsenic bioaccumulation

Internal As concentrations in earthworms (AsE) increased with increasing soil As concentrations (Table 2). In earthworms from control soils, average arsenic concentration was 32.8 (±16.3) μg As g^−1^ dry weight (*n* = 21). When exposed to 600 mg As kg^−1^ dry weight, the earthworms showed the highest internal concentration in soils H1, H2, H4 and H7, ranging from 1019 μg As g^−1^ in H7 to 525 μg As g^−1^ in H2, meanwhile soils H3 and H5 presented the highest AsE (762 and 768 μg As g^−1^, respectively) in the treatment of 300 mg As kg^−1^ dry weight. In soil H6, the maximum value of AsE was 740 μg As g^−1^ in the treatment of 100 mg As kg^−1^, and there were no survivors at higher exposure concentrations.

Biota-soil accumulation factor (BSAF) ranged between 0.93 and 7.67 kg soil kg^−1^ earthworm in control soils. While for the highest contamination level (600 mg As kg^−1^), values ranged between 0.87 and 1.93, with the exception of soils H3 and H6 in which there were no survivors for this treatment. In general, BSAF tended to decrease with increasing As concentration in soil (regardless of controls), except for soils H1 and H2, with no significant changes between treatments, and H7 where BSAF increased with increasing AsT (Table 2). Biota-water accumulation factor (BWAF) ranged between 258 and 9710 L kg^−1^ earthworm in the controls, while for the highest As treatment BWAF, values were ranged from 1.96 in H2 to 362 in H7 soils. In general, BWAF decreased with the increase in AsW in relation to the control, except for soils H4 and H7 in which the maximum BWAF values were found at the lowest As concentrations added (treatments of 50 and 100 mg As kg^−1^) (Table 2).

### Arsenic toxicity in earthworms

According to the OECD 222 guideline, the toxicity test is valid when earthworm mortality in the control is less than 10 % and more than 30 juveniles are produced in each replicate control. Based on this, all tests fulfilled these requirements except soil H1 for juvenile production, in which very low values were reached. Earthworm mortality (M) after 4 weeks was quite differently affected by arsenic contamination according to the different soil types, with less or equal than 7 % mortality in soils H2, less or equal than 20 % mortality in soils H4 and H7, and a dose-related decrease in soils H1, H3, H5 and H6. Mortality was only higher than 50 % in soils H3 and H6 (Table 3) at concentrations added of 300 mg As kg^−1^ soil.

**Table 3:** Mortality (M) was calculated as percentage of worms that died during the 4-week exposure period for each soil and treatment in relation to the control; weight variation (W) was calculated as the percentage of variation in the surviving earthworms recovered after 4 weeks in relation to the initial weight and recalculated in relation to the control soils; juvenile production (J) was calculated from the number of juveniles produced per worm per week and expressed as percentage in relation to the control

As nominal (mg kg^−1^)	H1			H2			H3			H4			H5			H6			H7		
	M	W	J	M	W	J^a^	M	W	J	M	W	J	M	W	J	M	W	J	M	W	J
0	0a	0.33a	100	3	0	100a	3a	0	100a	7	0b	100a	3ab	0a	100a	0a	0a	100a	10	0	100a
50	0a	−2.34a	150	0	−2.69	102a	3a	−8.20	70b	3	−15.37ab	91a	0a	9.32a	42b	33b	50.66ab	0b	3	13.59	74a
100	0a	−2.38a	217	3	3.71	230b	17a	−9.36	1.4c	17	0.91b	71b	3ab	3.72a	12c	50b	86.25b	0b	20	16.87	23b
300	3ab	12.75ab	0	0	0.36	238b	60b	18.40	0c	0	−28.63a	5.5c	23b	11.73a	0.1d	100c	ns	ns	10	10.53	0b
600	10b	22.26b	0	7	14.22	14a	97b	ns	ns	17	−16.02ab	0c	47c	41.64b	0d	100c	ns	ns	20	18.11	0b

Lowercase letters represent significance difference between treatments (Tukey HSD test. *p* <0.05). ns means not adult survival enough.

^a^Anomalous behaviour

The average individual earthworms weight in the controls at the beginning of the exposures was 501 ± 72 mg (±SD; *n* = 210), while after 4 weeks of incubation, it was 420 ± 65 mg. In the As-spiked soils, earthworm weights showed variation trends that were not always dose-related. In some cases, there was a trend of increasing earthworm weights in relation to the control (W) at higher As concentrations, which was significant for soils H1, H5 and H6. In the other soils, earthworm weight showed no significant dose-related variation compared to the control (Table 3).

Juvenile production (J) in relation to the control showed a decrease with increasing arsenic concentration for most studied soils. Juvenile reduction was significantly different from the control samples (*p* < 0.05) at the lowest treatment (50 mg As kg^−1^) for the soils H3, H5 and H6, and at the treatment 100 mg As kg^−1^ added for soils H4 and H7. Soil H2 showed an anomalous behaviour with the highest juvenile number in relation to the control at 100 and 300 mg As kg^−1^ soil (Table 3).

The results of the toxicity endpoints (lethal and effective concentrations) are shown in Table 4. For soils H2, H4 and H7, the arsenic concentrations used in this study in relation to earthworm survival (LC10) did not show toxicity response variation in relation to the control soils. LC10 values calculated from total As concentrations (AsT) ranged from 606 mg As kg^−1^ dry soil for H1 (close to the highest As concentration tested) to 31 mg kg^−1^ dry soil for H6. LC10 based on water-soluble As concentrations (AsW) was also highest for H1 soil (82 mg kg^−1^) and lowest for soil H6 with a value of 0.089 mg kg^−1^ (Table 4).

**Table 4:** Toxicity values for the effects of arsenic on the survival and reproduction of the earthworm *Eisenia andrei* after 4 and 8 weeks exposure, respectively, to different soils spiked with As

	Survival			Juveniles					
	LC10			EC50			EC10		
Soil	AsT (mg As kg^−1^ soil)	AsW (mg As kg^−1^ soil)	AsE (μg As g^−1^ worm)	AsT (mg As kg^−1^ soil)	AsW (mg As kg^−1^ soil)	AsE (μg As g^−1^ worm)	AsT (mg As kg^−1^ soil)	AsW (mg As kg^−1^ soil)	AsE (μg As g^−1^ worm)
H1	606	82	>793	ad	ad	ad	ad	ad	ad
	(476–736)	(46–118)							
H2	nd	nd	nd	ad	ad	ad	ad	ad	ad
H3	132	77	626	60	8.4	490	45	4.4	425
	(15–250)	(0–171)	(597–654)	(55–65)	(7.5–9.4)	(465–515)	(39–51)	(3.5–5.2)	(378–473)
H4	nd	nd	nd	151	1.2	495	82	0.11	366
				(130–172)	(0.6–1.8)	(421–569)	(63–100)	(0–0.23)	(194–537)
H5	188	12	734	56	0.84	584	26	0.19	497
	(57–318)	(0–25)	(702–766)	(48–63)	(0.66–1.03)	(567–601)	(15–37)	(0.06–0.32)	(454–540)
H6	31	0.089	356	nd	nd	nd	nd	nd	nd
	(12–49)	(0–0.186)	(105–606)						
H7	nd	nd	nd	96	0.013	84	59	0.006	46
				(80–111)	(0.008–0.017)	(70–98)	(38–79)	(0.002–0.009)	(29–63)

LC10 for effects on survival and EC50 and EC10 for effects on juvenile production were calculated using the total AsT and the water-soluble arsenic (AsW) in the soils and the internal arsenic concentrations (AsE) in the earthworms. AsT and AsW are expressed in milligrammes As per kilogramme dry soil and AsE as micrograms As per gramme earthworm. See Table 1 for soil properties and Table 3 for survival and reproduction data. Values in between brackets are 95 % confidence intervals. Values > (higher) than the highest treatment (600 mg kg^−1^) adding soil background

*ad* anomalous data, *nd* no dose-response observed

Earthworm reproduction based on juvenile production data was more sensitive than survival data. For soils H1, H2 and H6, no toxicity endpoints were calculated because no dose-related variation was observed. Soils H4 and H7 showed the lowest toxicity according to juvenile production for AsT, with an EC50 of 151 and 96 mg As kg^−1^, respectively; meanwhile, arsenic toxicity was highest in soil H5 with EC50 and EC10 values of 56 and 26 mg kg^−1^ (AsT), respectively. However, in relation to water-soluble concentrations (AsW), soil H3 was the least toxic (EC50 of 8.4 mg As kg^−1^), and soil H7 was the most toxic for juvenile production (EC50 of 0.013 mg As kg^−1^) (Table 4).

Toxicity endpoints were also calculated by the internal As concentrations (AsE) in the surviving earthworms. This variable showed lower differences between soils than the endpoints calculated from AsT and AsW. LC10 based on internal As concentrations was lowest in soil H6 (LC10 356 μg g^−1^ dry earthworm) and highest in H1 (LC10 > 793 μg g^−1^ dry earthworm). For juvenile production, EC50 related to AsE was lowest for soil H7, with values of 84 μg g^−1^ dry earthworm, and the highest value was for H5 soil (584 μg g^−1^ dry earthworm) (Table 4).

### As bioavailability and toxicity in relation to soil properties

Spearman correlation between the variables related to As bioavailability and toxicity (Table 5) showed that AsE was directly correlated with AsT and AsW, and inversely with Kp; meanwhile, biota-soil accumulation (BSAF) was only inversely correlated with AsT. Biota-water accumulation factor (BWAF) was inversely correlated with AsT, AsW and AsE, and directly with Kp. In relation to earthworm response, earthworm mortality (M) was directly related to AsT and AsE concentrations, and earthworm weight variation (W) was positively correlated with AsE and mortality. Juvenile production (J) showed a negative correlation with AsT, AsW and AsE and also with mortality and weight variation, while Kp was positively correlated with juvenile production.

**Table 5:** Correlation coefficients (Spearman) between total arsenic (AsT), water extractable As (AsW), partition coefficient (Kp), internal As concentrations in the earthworms (AsE), biota-soil accumulation factors (BSAF); biota-water accumulation factors (BWAF) and earthworm (*Eisenia andrei*) responses: mortality (M), weight variation (W) and juvenile production (J)

	Spearman correlations								
	AsT	AsW	Kp	AsE	BSAF	BWAF	M	W	J
AsT (mg As kg^−1^ soil)	1	0.777**	−0.581**	0.747**	−0.459**	−0.633**	0.431*		−0.647**
AsW (mg As kg^−1^ soil)		1	−0.957**	0.759**		−0.953**			−0.527**
Kp (L kg^−1^)			1	−0.645**		0.944**			0.399*
AsE (μg As g^−1^ earthworm)				1		−0.576**	0.533**	0.410*	−0.781**
BSAF (kg soil kg^−1^ earthworm)					1				
BWAF (L kg^−1^ earthworm)						1			
M (%)							1	0.551**	−0.734**
W (%)								1	−0.436**
J (%)									1

*p < 0.05; **p < 0.01

PCA with the toxicity endpoints and the main soil variables of this study showed that 81.3 % of the variance was explained by a total of five components (Table 6). The responses of the earthworm to As toxicity (mortality, weight variation and juvenile production) were grouped in component 2 with the As forms (AsT, AsW and AsE) with a direct relation in all cases except in juvenile production which showed an inverse relation with all these components. Component 1 grouped some of the main soil properties like organic carbon content (OC), available phosphorous content (P), amorphous Mn forms (Mn_o_) and cation exchange capacity (CEC). Component 3 grouped ionic strength (I) and amorphous iron forms (Fe_o_) together and inversely related with pHH_2_O; weight variation of the earthworms (W) as well as the AsW was also grouped in this component with positive and negative coefficients, respectively, but with a low load in this component. BSAF was included in component 4 and negatively related with AsT, CEC, clay content and amorphous Al forms (Al_o_). Finally, BWAF, Kp and clay content were included in component 5 with a positive relation.

**Table 6:** PCA of rotated component matrix (varimax with Kaiser normalization) for As studied forms, the toxicity earthworm endpoints and the main variables of the studied soils

	Components				
	1	2	3	4	5
AsT (mg As kg^−1^ soil)		0.711		0.507	
AsW (mg As kg^−1^ soil)		0.618	−0.561		
Kp (L kg^−1^)					0.859
AsE (μg As g^−1^ worm)		0.788			
BSAF (kg soil kg^−1^ earthworm)				−0.855	
BWAF (L kg^−1^ earthworm)					0.825
M (%)		0.781			
W (%)		0.539	0.502		
J (%)		−0.794			
pH (water)			−0.813		
I (mmol L^−1^)			0.628		
OC (%)	0.931				
CEC (cmol^+^ kg^−1^)	0.821			0.517	
Clay (%)				0.661	0.515
P (mg kg^−1^)	0.984				
Al_o_ (‰)				0.764	
Mn_o_ (‰)	0.913				
Fe_o_ (‰)			0.890		
% ac.ex.var	19.9	38.0	54.2	69.1	81.3

*% ac.ex.var.* percent of accumulated explained variance

## Discussion

According to the arsenic mobility in relation to soil properties and constituents, iron oxides have been widely described as the main active constituents determining As retention in soil (Fitz and Wenzel 2002), which coincides with our study where the lowest water-soluble As concentrations were found in the iron-rich soil (H7). Moreover, As distribution between the soluble and the solid phases is related also to organic carbon content (Yang et al. 2002). In our case, sample H1 and H5 (with the highest organic carbon contents) showed low values of AsW for the highest treatment (Table 2). A previous study with the same soils treated with the same As concentrations and conditions (Romero-Freire et al. 2014) confirmed the inverse correlation of AsW concentrations with OC content in the treatments with high concentrations of As added. Furthermore, it is known that the solubility of arsenic decreases when ionic strength increases (Acosta et al. 2011), and in our study, soils H6 and H4 (with the lowest ionic strength) presented low AsW concentrations in relation to the total As content. The highest AsW concentrations were found in the soils with the highest CaCO_3_ content and basic pH (H2 and H3), which is in accordance with other studies revealing that under certain conditions a higher pH may enhance As solubilization (Simon et al. 2010).

Partition coefficient (Kp) is crucial to estimate the potential for the adsorption of dissolved arsenic in contact with soil (USEPA 1999) and is strongly influenced by soil parameters. However, there is no consensus in literature to state these parameters due to it is obtained from a wide range of soil to water ratios (Sauvé et al. 2000), hindering the comparison among studies. Nevertheless, Kp is a key parameter to compare in our study soils under the same testing conditions and arsenic extraction method. Our study showed that Kp was directly correlated with iron and aluminium forms as well as ionic strength and clay content. Other studies (Song et al. 2006; Romero-Freire et al. 2014) showed that some of these properties play an important role in the As solubility which are strongly related to this coefficient. Sauvé et al. (2000) also reported some other soil properties like pH or OC as essentials to predict Kp values due to their influence on As solubility.

Internal arsenic concentrations in earthworms (AsE) varied between soils and increased with increasing exposure both AsT and AsW concentrations. AsE reported by other authors showed a wide range of values; Janssen et al. (1997) showed for *E. andrei*, in 20 different Dutch soils, a range from 2.99 to 65.2 μg As g^−1^ dry weight; García-Gómez et al. (2014) found for control soils an average of 18 μg As g^−1^ dry weight for *Eisenia fetida*; and Langdon et al. (2003) reported values between 3 μg As g^−1^ (soil total concentration 5 mg As kg^−1^) and 900 μg As g^−1^ (soil total concentration 87 mg As kg^−1^) based on a review for different species of earthworms. However, other authors showed lower concentrations. Peijnenburg et al. (1999) found in soils without arsenic pollution a mean tissue concentration of 3.75 μg As g^−1^ dry weight for *E. andrei*, and Beyer et al. (1985) gave an internal arsenic concentration of 5–6 μg As g^−1^ for *E. fetida*. In earthworms of the genus *Eisenoides*, Beyer and Cromartie (1987) found an internal concentration of 0.17–1.5 μg As g^−1^ dry weight, suggesting that different earthworm species show great differences in metal accumulation. Fischer and Koszorus (1992) defined the maximal accumulation capacity at 902 μg As g^−1^ dry weight for *E. fetida* at sub-lethal exposure concentrations in long-term studies, which is close to our findings (maximal accumulation capacity obtained 1019 ± 167 μg As g^−1^ dry weight); at higher body concentrations, all earthworms died.

BSAF and BWAF values showed a decrease with increasing exposure levels in most of the studied soils, suggesting that arsenic is mainly autoregulated by *E. andrei* in these soils. Such negative relationship is common for metals (McGeer et al. 2003) and was found for molybdenum (Díez-Ortiz et al. 2010) and also for the bioaccumulation of As in *E. fetida* exposed to a mine soil containing high As concentrations (García-Gómez et al. 2014). As a consequence, the highest BSAF and BWAF values usually occur at low background levels and decrease as pollution levels increase (Williams et al. 2006), which suggests some control over As bioaccumulation. However, in soil H7, BSAF only increased at the highest total As concentration (Table 2), and in soils H4, H5 and H6, BSAF values showed an increase compared to the control at different treatments of As added. These cases suggest the earthworms may be capable of sequestering arsenic, leading to higher body concentrations than expected, but it remains unclear what is the mechanism of arsenic sequestration. Fischer and Koszorus (1992) reported BSAF values for As in *E. fetida* between 18.1 and 10.3 upon exposure to soils polluted with 23–87 mg As kg^−1^ soil, which are higher than the BSAF values found in our study with *E. andrei*.

Otherwise, BWAF values decreased with increasing soil As concentrations, except for soils H4 and H7 (Table 2). In soil H4, BWAF was higher than that in the control at 50 and 100 mg As kg^−1^ dry soil, while in soil H7, this was the case for all treatments except for the highest one (600 mg As kg^−1^). These findings suggest that As concentrations in earthworms did not change proportionally with changes in water-soluble arsenic induced by increasing total As concentration in soil. US EPA (2007) also reported that for As, and other nonessential metals, accumulation is nonlinear with respect to exposure concentration. In general, BWAF values could be comparable to BCF due to both factors were calculated from the water-soluble arsenic. In this sense, the bioconcentration factor (BCF) proposed by the arsenic ambient water quality criteria (AWQC), calculated from the milligramme of As in a litre of water, is 44 L kg^−1^, derived from BCF values of 1 for fish and 350 for oysters (Williams et al. 2006); in this paper, the highest BCF values reported by other studies were 1600 and 3091 L kg^−1^ in different fish species. Otherwise, US EPA (2003) suggested a BCF range between 150 and 10,000 L kg^−1^ for different species. Results obtained in our study showed in general also high BWAF values in the range of the reported values. Our results and those obtained by other authors highlight the potential of As for bioaccumulation in food chains (McGeer et al. 2003; Williams et al. 2006), although the variation in BSAF and BWAF values in relation to the contamination levels indicates that these are no good indicators of potential environmental risks (García-Gómez et al. 2014; McGeer et al. 2003).

Earthworm mortality was correlated with AsE in our studied soils, although total or soluble arsenic concentrations in soil should better explain the mortality observed in earthworms than the body accumulation (Table 5). In fact, internal concentrations in earthworms do not necessarily correlate with the concentration at the site or the toxic action (Smith et al. 2012; García-Gómez et al. 2014). However, in some of our studied soils, mortality did not reach 50 % at the highest As concentration added (600 mg As kg^−1^ soil), which reflects the importance of soil properties in reducing As availability and therefore toxicity. Mortality also was related to earthworm weight, with a significant trend to increasing earthworm weights at the highest As concentrations in soils H1, H5 and H6. This suggest that only the biggest earthworms survived arsenic exposure, although it might also be possible that surviving earthworms were more tolerant to As and benefitted from the reduced density and resulting higher food availability to grow bigger. Some authors suggest that earthworm weight loss could be caused primarily by soil factors, especially with soil pH, while body metal concentrations play a minor role (Janssen et al. 1997). In our study, weight variation appeared inversely related with pHH_2_O (Table 6), as well as it was correlated with body As concentrations, finding the highest mortality in sample H6, soil with the lowest pH (Table 3). Moreover van Gestel et al. (1992) found that the growth of *E. andrei* was negatively related with reproduction, which also was found in this study (Table 5). Reproduction was more sensitive to As than survival. A large variation in juvenile production was found in the different tested soils, which could be due to the variation in soil properties (van Gestel et al. 1992). According to the OECD guideline 222, the requirements for the control sample need that the earthworm mortality should be less than 10 % and juvenile production higher than 30 juveniles. Control reproduction was good in all samples with the exception of H1 soil, being that all earthworms were taken from the same batch, it must be concluded that the low reproduction in soil H1 was due to the unfavorable properties of the soil rather than to problems with the health of the tested animals. Juvenile production was negatively correlated with total and water-soluble As concentrations in the soils and with internal concentrations in the earthworms. Reproduction was lowest in soil H7, having a high clay content (which could affect water availability) and a high aluminium content, that could have influenced in the reduction of growth and juvenile production (van Gestel and Hoogerwerf 2001). Optimal environmental conditions for the reproduction of *E. andrei* are high OC content, pH (CaCl_2_) between 4.5 and 6.5, and a moisture content of 50 % approximately of the maximum soil water holding capacity (van Gestel et al. 1992). Control conditions in soil H5, with a pH-KCl of 6.7 and high organic carbon content, probably were most favourable, explaining the high number of juveniles produced in this soil, with an average of juveniles per worm per week of 6 ± 0.6.

The reproduction of *E. andrei* was more sensitive to arsenic than the other studied parameters; thus, the obtained toxicity endpoints could be considering more accurate. Root elongation test performed with *Lactuca sativa* on the same soils (Romero-Freire et al. 2014) showed similar EC10 values for total As compared with *E. andrei* reproduction in soils H4 and H5 (95 mg kg^−1^ and 38 mg kg^−1^ soil values for lettuce, respectively). Soil H3 showed a more restrictive EC10 for lettuce (23 mg kg^−1^ soils) than for earthworms, with values differing by a factor of two. In soil H7, lettuce was less sensitive (395 mg kg^−1^), while earthworm reproduction gave an EC10 of 59 mg kg^−1^ in the same soil. These results highlight the influence of soil properties on arsenic bioavailability and therefore toxicity and also the importance of selecting different organisms when defining guideline values for ERA.

The study of the influence of soil properties in As toxicity for earthworm showed controversial results. Other studies with different pollutants showed pH, CEC, Ca content, Mn oxides, OC content, clay and silt content as the most relevant factors affecting earthworm toxicity for metals (van Gestel et al. 2011; Bradha et al. 2006; Peijnenburg et al. 2002; Janssen et al. 1997). However, our results showed that earthworm mortality in different As-treated soils was greatly explained by As soil concentration as well as internal concentration in earthworms and low influence of soil properties was found. Earthworm weight variation, also related with the studied forms of As, was observed that could increase with the rise in I strength of the soils as well as could be indirectly related with pH (highest pH lower weight variation). While juvenile production in our study decreased with higher mortality or earthworm loss body weight, the ionic strength, the pH and the presence of iron may be considered as soil properties which could have a role in earthworm answer to As toxicity. Our study suggested that the influence of soil properties in As solubility and availability indirectly control the influence As toxicity in earthworms. However, related to other bioassays performed with lettuce and a marine bacteria (*V. fischeri*) (Romero-Freire et al. 2014), soil properties had a direct influence in their toxicity answer. In this sense, As behaviour in soils and the complex pathways of incorporation in body oligochaetes should be further studied to propose robust generic environmental quality standards.

Finally, in this study, we worked with soils contaminated with concentrations chosen according to the proposed values for Andalusia representing the criteria to declare a soil as contaminated for different soil uses (Aguilar et al. 1999). The selection of these values was based on data levels proposed by other countries. However, these values do not take into account the difference in soil types. Otherwise, laboratory-spiked soils instead of field-contaminated soil were used in this study because spiked soils tend to overestimate the availability of metals in field soils (Smolders et al. 2009); therefore, the toxicity level defined can provide with more certainty the safety threshold for the environmental risk assessment (ERA). It is therefore necessary to further investigate mobility and bioavailability of As in the ecosystem in relation to soil properties to propose reference values useful in the declaration of contaminated areas.

## Conclusions

Arsenic solubility and therefore availability were largely explained by soil properties and constituents such as pH, calcium carbonate content, oxides forms, clay content and ionic strength of the soil solution and should be considered essential parameters influencing arsenic toxicity in soils. Internal arsenic concentration (AsE) in the studied earthworms (*Eisenia andrei*) increased directly with As solubility in soils which highlight the potential of As for bioaccumulation in food chains. Moreover, AsE varied between soils suggesting that earthworms could be able to sequester arsenic. Further studies to deepen in the relationships between As toxicity of earthworms and soil properties should be performed. The results of the present study in relation to the earthworm answer against As toxicity indicated that earthworm survival, body weight variation and earthworm reproduction are strongly influence by solubility of arsenic as well as AsE; therefore, soil properties could greatly reduce indirectly As bioavailability and toxicity risk for the studied oligochaeta. The comparison of the obtained results with other different organisms against As toxicity showed a high variability and therefore indicated the importance of selecting different organisms when defining guideline values for ERA.
